# Assessment of the Effects of Low-Level Laser Therapy on the Thyroid Vascularization of Patients with Autoimmune Hypothyroidism by Color Doppler Ultrasound

**DOI:** 10.5402/2012/126720

**Published:** 2012-12-17

**Authors:** Danilo Bianchini Höfling, Maria Cristina Chavantes, Adriana G. Juliano, Giovanni G. Cerri, Meyer Knobel, Elisabeth M. Yoshimura, Maria Cristina Chammas

**Affiliations:** ^1^Ultrasound Unit, Department of Radiology, Clinics Hospital, University of Sao Paulo Medical School, 05403-000 São Paulo, SP, Brazil; ^2^Laser Medical Center, Heart Institute, Clinics Hospital, University of Sao Paulo Medical School, 05403-000 São Paulo, SP, Brazil; ^3^Thyroid Unit, Department of Endocrinology and Metabolism, Clinics Hospital, University of Sao Paulo Medical School, 05403-000 São Paulo, SP, Brazil; ^4^Department of Nuclear Physics, Physics Institute, University of Sao Paulo, 05508-900 São Paulo, SP, Brazil

## Abstract

*Background*. Chronic autoimmune thyroiditis (CAT) frequently alters thyroid vascularization, likely as a result of the autoimmune process. *Objective*. To evaluate the effects of low-level laser therapy (LLLT) on the thyroid vascularization of patients with hypothyroidism induced by CAT using color Doppler ultrasound parameters. *Methods*. In this randomized clinical trial, 43 patients who underwent levothyroxine replacement for CAT-induced hypothyroidism were randomly assigned to receive either 10 sessions of LLLT (L group, *n* = 23) or 10 sessions of a placebo treatment (P group, *n* = 20). Color Doppler ultrasounds were performed before and 30 days after interventions. To verify the vascularity of the thyroid parenchyma, power Doppler was performed. The systolic peak velocity (SPV) and resistance index (RI) in the superior (STA) and inferior thyroid arteries (ITAs) were measured by pulsed Doppler. *Results*. The frequency of normal vascularization of the thyroid lobes observed in the postintervention power Doppler examination was significantly higher in the L than in the P group (*P* = 0.023). The pulsed Doppler examination revealed an increase in the SPV of the ITA in the L group compared with the P group (*P* = 0.016). No significant differences in the SPV of the STA and in the RI were found between the groups. *Conclusion*. These results suggest that LLLT can ameliorate thyroid parenchyma vascularization and increase the SPV of the ITA of patients with hypothyroidism caused by CAT.

## 1. Introduction

Chronic autoimmune thyroiditis (CAT) is the most common cause of hypothyroidism in populations who have an adequate dietary iodine intake [1]. The combination of genetic and environmental factors may contribute to the aetiology of the CAT [2, 3]. The autoimmune responses mediated by cells, antibodies and cytokines promote thyroid follicular cell injury in CAT patients [3–5]. This disease leads to the progressive destruction of the follicular cells of the thyroid parenchyma, which can result in hypothyroidism.

Recent evidences have suggested that low-level laser therapy (LLLT) may improve thyroid function and reduce the levels of thyroid peroxidase antibodies (TPOAb) in patients with hypothyroidism caused by CAT [6, 7]. Color Doppler ultrasound (CDU) provides important additional information beyond that obtained by B-mode ultrasound, allowing the evaluation of various parameters of thyroid vascularization.

The presence of increased vascularization in the thyroid parenchyma in most CAT patients has been demonstrated in recent studies [8, 9]. It was also reported that the systolic peak velocity of the thyroid arteries may be increased in patients with autoimmune diseases of the thyroid gland, including CAT [10].

The thyroid is a superficial gland, and a laser can easily access it transcutaneously. The anti-inflammatory effects of LLLT on organic tissues have been shown in several studies [11, 12]. The initial results of LLLT in patients with hypothyroidism promoted by CAT suggested that there may be an improvement in thyroid function, a reduction in thyroid antiperoxidase antibody level and an increase in the thyroid parenchymal echogenicity as assessed by B-mode ultrasound. Because the abnormal thyroid vascularization of CAT patients likely results from the autoimmunity present in the gland [8, 10], it is possible that the anti-inflammatory effects of LLLT may improve the vascular parameters evaluated by CDU.

Thus, the objective of this study was to evaluate the effects of LLLT on the thyroids of patients with CAT-induced hypothyroidism by evaluating CDU parameters.

## 2. Material and Methods

### 2.1. Subjects

Patient selection was carried out between March 2006 and January 2008 in the Thyroid Outpatient Clinic of the Hospital das Clínicas, University of Sao Paulo Medical School (HC-FMUSP). This study was conducted at the Radiology Institute of the HC-FMUSP, where most of the population is exposed to a sufficient intake of iodine [13].

In the Thyroid Outpatient Clinic, 108 patients with established hypothyroidism caused by CAT who were most likely to be eligible for inclusion in the trial were initially selected. These patients were undergoing treatment with stable doses of at least 50 *μ*g/day levothyroxine (LT_4_).

### 2.2. Inclusion Criteria

Patients who were between the ages of 20 and 60 years old, who had been undergoing LT_4_ therapy for hypothyroidism that was induced by CAT, and who had normal serum levels of triiodothyronine (T_3_), tetraiodothyronine (T_4_), free-T_4_(fT_4_), and thyrotropin (TSH) were selected for the study. The appropriate dose of LT_4_ replacement for each patient was determined by the doctors of the Thyroid Outpatient Clinic.

The CAT diagnosis was defined by the presence of the following criteria: (1) high serum levels of thyroid peroxidase antibodies (TPOAbs) and/or antithyroglobulin (TgAbs) antibodies (>100 U/mL); (2) overt hypothyroidism that required LT_4_ replacement and (3) an ultrasound pattern compatible with CAT [14–16].

### 2.3. Exclusion Criteria

Exclusion criteria included: (1) the use of immunosuppressants, immunostimulants, and drugs that interfere with the production, transport, and metabolism of thyroid hormones (e.g., corticosteroids, lithium, and amiodarone); (2) CAT with normal thyroid function; (3) CAT with subclinical hypothyroidism; (4) thyroid nodules; (5) hypothyroidism stemming from postpartum thyroiditis (up to 18 months after gestation); (6) a history of Graves' disease; (7) prior treatment with radioiodine; (8) tracheal stenosis; (9) pregnancy; (10) a history of exposure to ionizing radiation and/or neoplasia in the cervical area; (11) previous surgical intervention in the thyroid; (12) thyroid hypoplasia; (13) ectopic thyroid; (14) serious illness (e.g., cancer, ischemic coronary artery disease, stroke, and kidney or liver failure).

**Figure 1:**
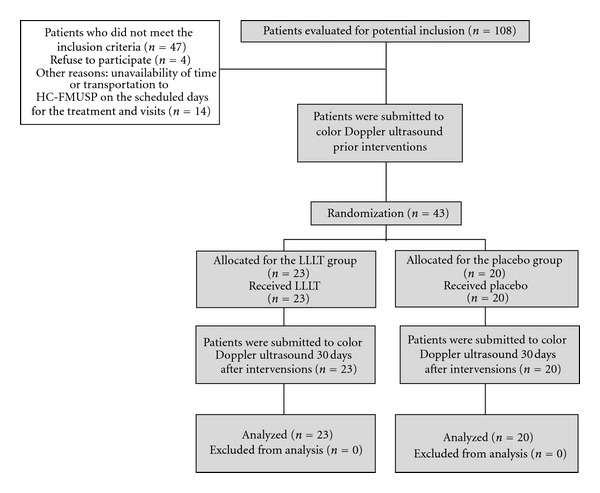
Flow diagram of the study protocol.

Of the 108 patients who initially presented with a high probability of inclusion, 47 were subsequently excluded because they did not fully meet the eligibility criteria, 14 lacked the time or transportation to get to the hospital, and 4 declined to participate (Figure 1). In the end, 43 patients were included in the study.

HC-FMUSP's Research Ethics Committee approved this study and the patient consent forms. All of the patients willingly signed the consent form.

### 2.4. Study Design

We designed a randomized, placebo-controlled clinical trial to evaluate the effects of LLLT on the thyroid glands of CAT patients by employing CDU variables. A computerized random number generator was used to individually assign patients to either the LLLT group (L) or the placebo group (P), and the resulting computer-generated codes corresponding to the group assignments were placed in individual opaque envelopes numbered from 1 to 60. The envelopes were only opened by the investigator responsible for the interventions (Höfling, DB) at the time of each patient's first therapy procedure; this practice guaranteed that the investigator was unaware of the group to which each patient was assigned until the envelope was opened. This researcher became aware of the patient assignments only after the allocation of patients to each group because it was necessary to adjust the equipment to administer either LLLT or the placebo. Thus, except for this investigator and the statistician, all of the patients, the Thyroid Outpatient Clinic physicians (who adjusted the appropriate dose of LT4 replacement), the laboratory personnel, and the CDU researcher were blinded to the treatment assignments for the duration of the study.

Ultrasound was used to mark the thyroid limits on the skin with a dermographic pen. Next, we created a mask (mold) for each patient using an adhesive plastic material that displayed the following demarcations: the gland contours (containing perforations of 3 mm in diameter separated by 1 cm on the inside), the jugular notch of the sternum, and the thyroid cartilage prominence; these latter 2 structures were easy to detect, which made the placement of the mask at the exact location of the thyroid simple and reproducible. The device tip was placed perpendicular to the skin in contact mode through the perforations located within the thyroid boundaries of the mold.

**Figure 2:**
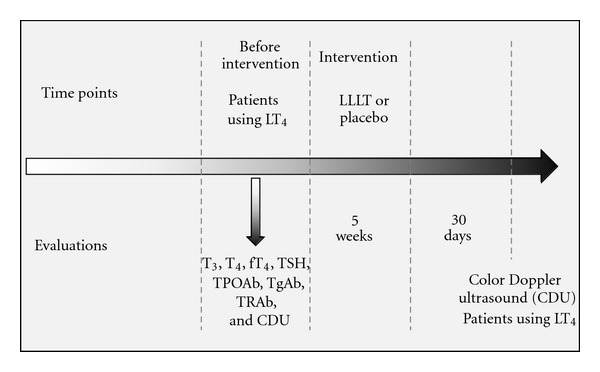
Study design.

Both groups underwent ten sessions of either LLLT or placebo treatment twice a week for 5 weeks (Figure 2). The laser equipment was calibrated before each procedure.

The L group was treated with a continuous-wave diode laser device (830 nm, infrared) with a beam area of 0.002827 cm^2^ (Theralase, DMC, São Carlos, SP, Brazil) using the punctual method in the continuous emission mode at an output power of 50 mW and at a fluence of 707 J/cm^2^ (40 seconds at each application point). Thus, the total energy deposited at each point was 2 J. The P group was treated using the same method and equipment, except an ordinary red light that was indistinguishable from the laser beam that was used as a placebo; this approach blinded the patients to which treatment they received.

### 2.5. Biochemical Measurements

The serum levels of TSH, T_3_, T_4_, fT_4_, TPOAb, and TgAb were measured using AutoDELFIA kits (Wallac Oy and PerkinElmer, Turku, Finland) prior to the intervention (Figure 2). Serum TRAb levels were determined prior to treatment (Figure 2) using a radio-receptor assay (RSR, Cardiff, UK). The reference values, analytical sensitivities, intraassay coefficients of variations and inter-assay coefficients of variations for the aforementioned assays are the following, respectively: (1) T_3_ = 70–200 ng/mL, 20 ng/dL, 2.9% and 1.2%; (2) T_4_ = 4.5–12.0 *μ*g/dL, 0.39 *μ*g/dL, 2.7% and 1.4%; (3) fT_4_ = 0.7–1.5 ng/dL, 0.16 ng/dL, 1.2% and 3.7%; (4) TSH = 0.4–4.5 *μ*U/mL, 0.01 *μ*U/mL, 2.8% and 2.2%; (5) TPOAb < 35 U/mL, 1.0 U/mL, 3.8% and 4.8%; (6) TgAb < 35 U/mL, 1.0 U/mL, 3.1% and 8.8%; (7) TRAb < 8.0%, 8%, 6.8%, and 9.6%.

### 2.6. Color Doppler Ultrasound Study

All of the CDU examinations were performed by a single-blinded investigator. CDU was performed using an HDI-5000 device (Philips Medical Systems, Bothell, WA, USA) attached to a broadband linear probe (5–12 MHz). CDU examinations were performed on patients who were using the same doses of LT_4_ at before intervention and 30 days after intervention (Figure 2).

### 2.7. Examination Technique

The CDU study was performed with the patient in the supine position and a cushion under their shoulders with their neck hyperextended. To avoid underestimating the vascularization intensity, the probe was lightly positioned on the skin without any compression. The CDU images were obtained using power and pulsed Doppler imaging.

### 2.8. Power Doppler

A short apnea was requested from the patients during the power Doppler recordings. The equipment was set to “thyroid” and configured as follows: pulse repetition frequency (PRF) between 700 and 1000 Hz, Map 1, Med, wall filter (WF) and flow opt: Med V. The probe was positioned in the longitudinal direction with its center at the projection of the middle third of the thyroid lobes. The isthmus vascularization was not assessed. The color gain was adjusted up to the highest possible level that was not associated with artifacts of image saturation. All of the two-dimensional images were recorded at the time of greatest visible flow, corresponding to the systolic peak velocity of blood flow.

**Figure 3:**
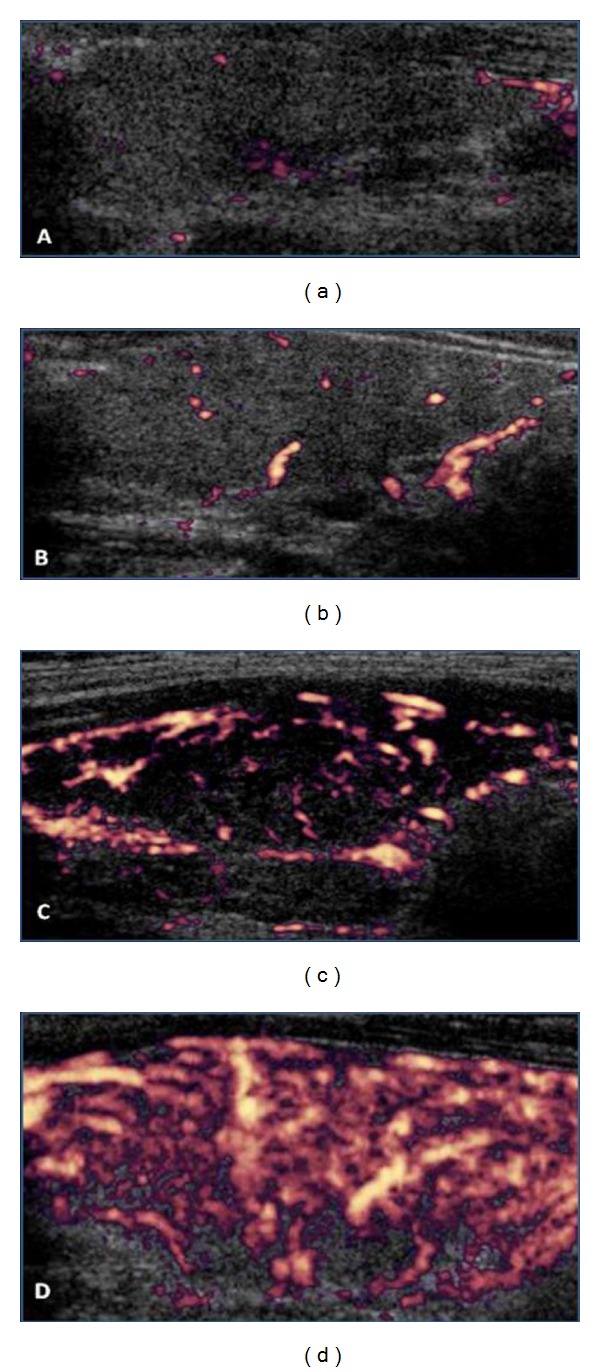
Power Doppler. Figures (a), (b), (c), and (d) correspond, respectively, to the vascularization patterns I, II, III, and IV of the thyroid parenchyma.

For the power Doppler analysis, the parenchyma vascularization was divided into four categories. Pattern I: the vascularization is decreased and is limited to the main peripheral arteries, which have reduced signals (assigned value = 0; Figure 3(a)). Pattern II: the vascularization is limited to the main peripheral thyroid arteries, which exhibit the usual signals, whereas there is either no flow signal in the parenchyma or only signals of vascularization focal points with either a scattered distribution (assigned value = 1; Figure 3(b)). Pattern III: clearly increased vascularity with a scattered distribution (assigned value = 2; Figure 3(c)). Pattern IV: a marked increase in vascularization with a diffuse and homogeneous distribution, including the so-called “thyroid inferno” pattern [17] (assigned value = 3; Figure 3(d)).

We assigned vascularization patterns from I to IV to the right and left lobes of each patient's thyroid, with 46 lobes in the L group and 40 lobes in the P group.

### 2.9. Pulsed Doppler

The sampling volume was set to 2 mm. The PRF was set according to the speed of the flow and to the parameters that yielded the best possible graphic representation. The evaluation of the superior thyroid artery was accomplished with the probe positioned in the oblique sagittal plane, close to the superior thyroid pole. The inferior thyroid artery was examined in the oblique axial plane, close to the transition between the middle and the inferior third of the thyroid. For the evaluation of the inferior thyroid artery, the cursor was set close to the trachea to avoid artifacts coming from the common carotid artery and the internal jugular vein. The systolic peak velocity and resistance index (RI) values in the superior and inferior thyroid arteries were obtained from pulsed Doppler analysis. The Doppler angle was always corrected to values less than or equal to 60°. The mean of the values found in the right and left lobes was used as the representative parameter.

### 2.10. Statistical Analysis

The statistical analysis was conducted primarily using SPSS software (version 16.0—SPSS Inc., IBM Headquarters Company, Chicago, IL, USA). The results were compared by paired and unpaired Student's *t*-tests for normally distributed data and Mann-Whitney tests for data that departed substantially from a normal distribution. Fisher's exact tests were used to analyze the categorical data. The results of the continuous variables are presented as the means ± confidence interval (CI). Two-sided *P* values <0.05 were considered statistically significant (*).

## 3. Results

Forty-three patients with hypothyroidism caused by CAT were enrolled in the study and randomized to receive either LLLT (L group, *n* = 23) or placebo treatment (P group, *n* = 20). No patients dropped out during the study period (Figure 1). No significant differences between the randomization arms were found in any of the key variables (Table 1).

All 43 randomized patients, underwent 10 sessions of either LLLT or placebo treatment, were subjected to CDU pre- and 30 days after intervention and were included in the final statistical analysis (Figure 1).

### 3.1. Color Doppler Ultrasound

Based on the preintervention power Doppler study, the thyroid vascularization was abnormal (increased or decreased) in 22/23 (95.65%) patients in the L group and in 17/20 (85.00%) patients in the P group. In the L group, 19/23 (82.61%) exhibited hypervascularization, 3/23 (13.04%) exhibited reduced vascularization, and only 1/23 (4.35%) exhibited normal vascularization of the thyroid parenchyma. In the P group, 14/20 (70.00%) exhibited hypervascularization, 3/20 (15.00%) exhibited reduced vascularization, and 3/20 (15.00%) exhibited normal vascularization of the thyroid parenchyma.

The frequency of normal vascularization of the thyroid lobes observed in the postintervention CDU study was significantly higher in the L than in the P group (*P* = 0.023; Table 2).

The assessment of the numerical values attributed to the vascularization was performed by paired and unpaired analyses. The paired analysis of the power Doppler study in the L group showed no statistically significant difference pre- and post-LLLT for the average value attributed to the vascularization pattern. In this group, the average value approached 1, which corresponds to normal vascularization. In contrast, a statistically significant increase (*P* = 0.015) for the average value attributed to the vascularization pattern was found in the P group, which distanced itself from the normal value 1 (Table 3).

The unpaired analysis between groups indicated that the average value attributed to the vascularization pattern after intervention was significantly lower in the L group and closer to 1 (the normal value) compared with the P group (*P* = 0.033; Table 1).

**Figure 4:**
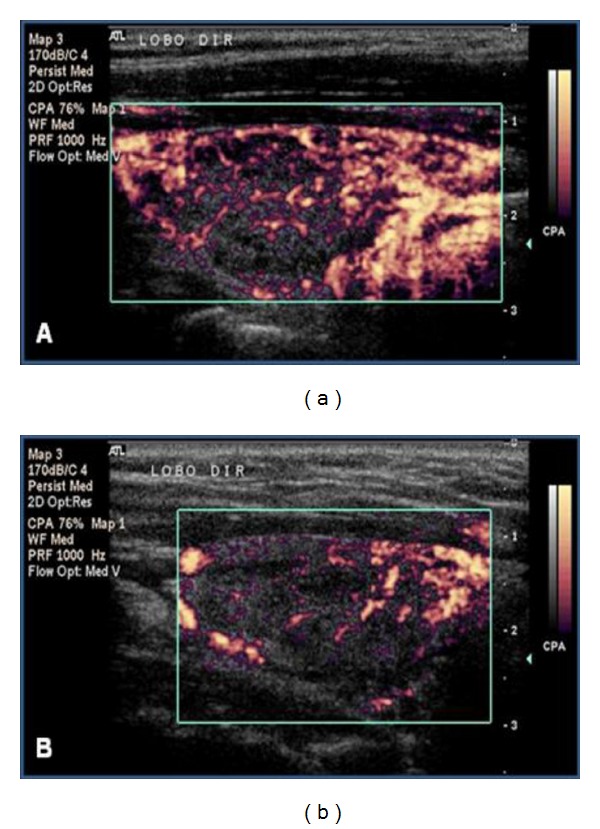
Thyroid parenchyma vascularization in an L-group patient in whom patterns III pre-LLLT (a) and II post-LLLT (b) were observed.

**Figure 5:**
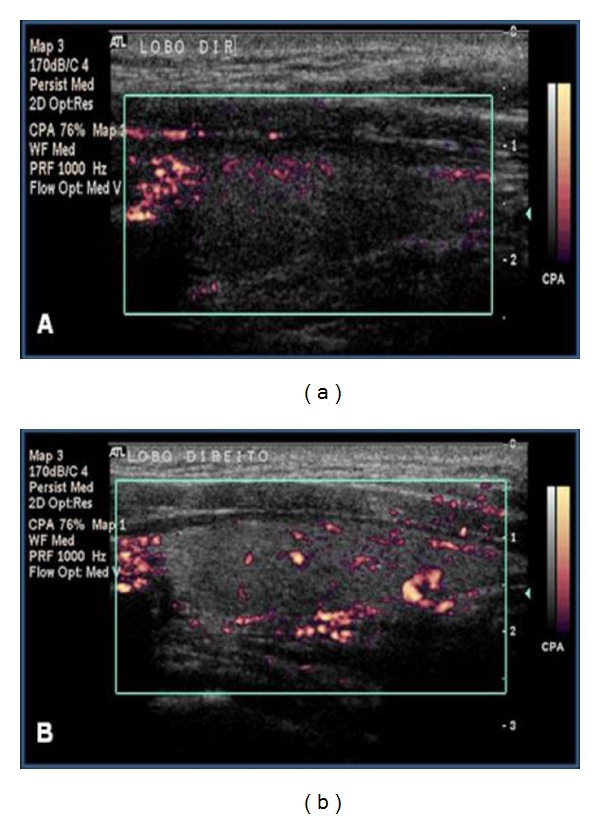
Power Doppler. Thyroid parenchyma vascularization in an L-group patient in whom patterns I pre-LLLT (a) and II post-LLLT (b) were observed.

**Figure 6:**
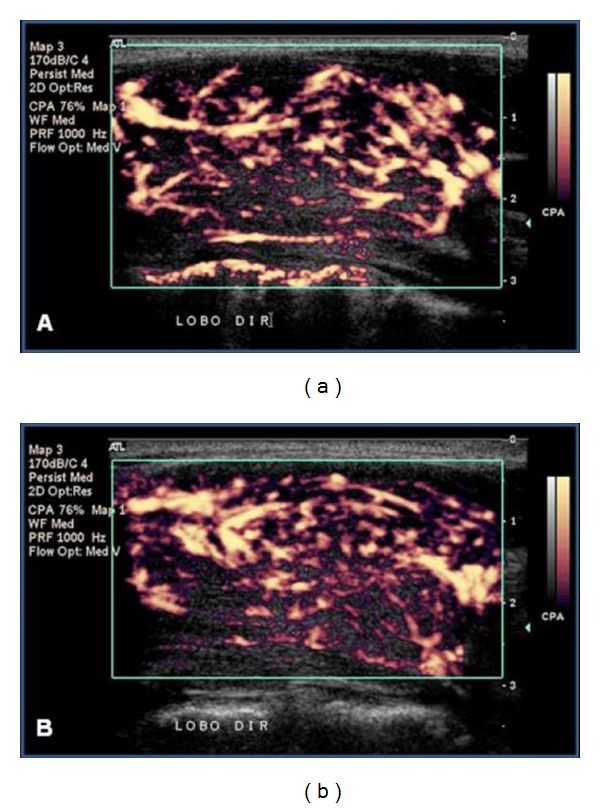
Power Doppler. Thyroid parenchyma vascularization in an L-group patient in whom the maintenance of pattern III pre-LLLT (a) and post-LLLT (b) was observed.

The pulsed Doppler analysis showed an increase in the systolic peak velocity of the inferior thyroid arteries in the L group compared to the P group (*P* = 0.016; Table 1), whereas the systolic peak velocity of the superior thyroid arteries was not significantly different between the groups. The mean RI also did not reveal significant differences between the L and P groups (Table 1). Figures 4, 5, and 6 present examples of the vascularization patterns observed pre- and postintervention.

## 4. Discussion

CDU was used to evaluate the effects of LLLT on the vascularization parameters of the thyroid gland, which are often altered in patients with TCA.

The LLLT demonstrated anti-inflammatory properties and the ability to regenerate biological tissues [11, 12, 18]. It was revealed that these effects of infrared lasers may be associated, at least in part, with changes in the gene expression and serum levels of several cytokines, such as TNF-*α*, IL-1*β*, IL-2, IL-6, IL-8, interferon-*γ* (IFN-*γ*) and TGF-*β* [18–21]. Elevated levels of proinflammatory cytokines, such as IFN-*γ*, TNF-*α*, IL-2, and IL-6, as well as the reduction of TGF-*β* levels, may play a role in CAT pathogenesis [22–24]. There are reports suggesting that LLLT can improve thyroid function, reducing the intensity of the autoimmune process against the gland as well as regenerating the follicle structure [6, 7]. It is possible that such LLLT effects may be mediated by cytokine modulation [7].

Parenchymal hypervascularization, which was observed in most of the patients in both the L and P groups prior to the intervention, is frequently found in CAT patients [8, 9]. The comparison of the vascularization pattern between the 2 groups after the intervention showed that the average value attributed to the vascularization was significantly lower in the L group, that is, closer to the normal value of 1 (Table 1). The opposite effect was observed in patients in the P group, that is, their values were significantly increased from the normal value. This result suggests that LLLT contributed to the improvement of abnormal vascularization (increased or decreased) in the vast majority of patients in the L group. Indeed, after intervention, the proportion of patients with normal vascularization was greater in the group that was subjected to LLLT.

High levels of TSH and TRAb could increase vascularization by elevating the levels of vascular endothelial growth factors in goitrous hypothyroidism and Graves' disease [9]. However, the TRAb serum levels were undetectable in all of the patients, and the levothyroxine dose was maintained until the completion of the postintervention CDU to maintain normal serum TSH levels.

It has been shown that the parenchymal thyroid vascularization is not the result of an increased production of thyroid hormones or TSH but rather of the autoimmune activity in the gland [8, 10, 25]. The increased autoimmune-induced thyroid vascularization could be explained, at least in part, by the increased serum levels of C-X-C motif chemokine 10 (CXCL-10), which is stimulated by the cytokine interferon-*γ* (IFN-*γ*), which is, in turn, involved in the recruitment of Th1 lymphocytes [8]. Newly diagnosed autoimmune thyroiditis patients demonstrate increased serum CXCL-10 levels [26]. A significantly higher serum level of CXCL-10 was also described in patients with increased thyroid vascularization [8]. The presence of higher CXCL-10 serum levels is more common in patients with CAT than in healthy individuals, and even after the correction of hypothyroidism by levothyroxine, there is no significant reduction of CXCL-10 [27]. This result suggests that the high CXCL-10 levels are associated with the intensity of the autoimmune process and not with hypothyroidism [27, 28].

The increased thyroid vascularization observed in most patients in this study, even in those undergoing treatment with levothyroxine, supports the hypothesis that the increased vascularization that is often present in CAT is independent of the serum TSH [8] and may be associated, at least in part, with the increasing levels of chemokine CXCL-10 [8, 27]. Considering that LLLT effects have been reported on several cytokines, it is plausible that this treatment may mediate, among others, the action of CXCL-10.

In individuals with no gland dysfunction, the SPV in the superior thyroid artery is significantly higher than that in the inferior thyroid arteries [29]. In this study, the preintervention SPV of the superior thyroid arteries was slightly higher than that of the lower arteries for both groups, although this difference was not statistically significant. However, the present study evaluated a smaller sample of patients with CAT than the cited study [29].

Before the intervention, the SPV and RI of the superior and inferior thyroid arteries were similar between the L and P groups. After the intervention, the SPV and RI of the superior thyroid arteries showed no significant differences between the two groups. In contrast, the inferior thyroid arteries showed a significant SPV increase in the L group compared with the P group. The RI of these arteries showed no significant difference between the two groups. This result cannot be attributed to high levels of TRAb and TSH or to an increase in thyroid hormones in the L group because no patients had detectable levels of TRAb, and there was no change in the levothyroxine replacement doses at the times of CDU execution, either before or after the intervention. In our pilot study [6], the increase in the SPV of the inferior arteries was close to statistical significance after LLLT. This outcome could be associated with the LLLT effects on chemokines or other cytokines and vascular growth factors related to gland vascularization. Further studies could investigate the possible effects of LLLT on the gene expression of cytokines in the thyroid tissue to clarify this result.

The increased SPV in the thyroid arteries is common in Graves' disease; however, patients with hypothyroidism caused by CAT (who receive treatment either with or without levothyroxine) can also exhibit this increase [10]. It has also been demonstrated that increases in SPV exist in the inferior thyroid arteries, even in cases of subclinical thyroid dysfunction, independent of serum TSH levels and thyroid hormones [25].

In summary, both the presence of diffuse parenchymal hypervascularization and the increase in SPV of the thyroid arteries, as assessed by pulsed Doppler ultrasound, can be found in patients with hypothyroidism caused by CAT [10]. The results of the abovementioned studies [8, 10, 25] indicate that the intensity of the color signals and the speed of the blood flow are probably the results of autoimmune activity, which is at least partly associated with the action of cytokines.

Thus, the results of this study suggest that LLLT was able to improve thyroid parenchyma vascularization, which is associated with the autoimmunity that is present in the glands of CAT patients. This improvement could be an indirect result of thyroid autoimmunity reduction. Moreover, an SPV increase in the inferior thyroid arteries was found in these patients, and we speculate that this result may be due to the action of LLLT on chemokines. The significance of this finding is, however, uncertain. It should be noted that this study evaluated the CDU parameters one month post-LLLT; therefore, it will be important to assess the duration of the LLLT results on the thyroid vascularization. Such findings corroborate those reported in our previous studies [6, 7], and the authors hope to encourage further studies on this subject.

In conclusion, the study of the thyroid gland using CDU showed that LLLT improved gland vascularization and increased the systolic peak velocity of the inferior thyroid arteries in patients with CAT-induced hypothyroidism.

## Figures and Tables

**Table 1:** Summary of the statistical analysis conducted for the trial groups: unpaired tests.

	Before-intervention			After-intervention		
Variables	L group *n* = 23, mean	P group *n* = 20, mean	*P* value^●^	L group *n* = 23, mean	P group *n* = 20, mean	*P* value^●^
	(CI of 95%)	(CI of 95%)		(CI of 95%)	(CI of 95%)	
Thyroid parenchyma vacularization	2.00 (1.56–2.44)	1.75 (1.29–2.21)	0.790	1.87 (1.51–2.23)	2.30 (2.03–2.57)	0.033*
Systolic peak velocity in the superior thyroid arteries (cm/s)	31.04 (26.12–35.96)	27.16 (22.79–31.53)	0.261	33.39 (27.33–39.45)	27.77 (23.61–31.93)	0.153
Systolic peak velocity in the inferior thyroid arteries (cm/s)	28.34 (23.60–33.08)	27.31 (23.22–31.40)	0.738	34.47 (29.66–39.28)	26.12 (21.83–30.41)	0.016*
Resistance index of the superior thyroid arteries	0.57 (0.54–0.60)	0.60 (0.56–0.65)	0.218	0.59 (0.56–0.62)	0.60 (0.57–0.63)	0.633
Resistance index of the inferior thyroid arteries	0.56 (0.53–0.59)	0.58 (0.54–0.61)	0.423	0.57 (0.53–0.61)	0.59 (0.56–0.61)	0.351

LLLT: low-level laser therapy; CI: confidence interval; cm/s: centimeter per second; ^●^: nonpaired Student's *t*-tests were used for compare the results between groups before- and after intervention; *: *P* < 0.05.

**Table 2:** Comparison of the observed frequencies of normal or abnormal vascularization determined by post-intervention color Doppler ultrasound in the L and P groups.

Vascularization	Post-LLLT *n* (%)	Postplacebo *n* (%)	Total *n* (%)	*P* value^●^
Normal vascularization	16 (18.6)	5 (5.8)	21 (24.4)	
Abnormal vascularization (increased or decreased)	30 (34.9)	35 (40.7)	65 (75.6)	

Total	46 (53.5)	40 (46.5)	86 (100)	0.023*

LLLT: low-level laser therapy; ^●^: Ficher's exact test at after intervention;

*: *P* < 0.05.

**Table 3:** Statistical analysis of power Doppler ultrasound results for the trial groups: paired tests.

	Pre-LLLT	Post-LLLT		Preplacebo	Postplacebo	
Variable	L group, *n* = 23	L group, *n* = 23	*P* value^●^	P group, *n* = 20	P group, *n* = 20	*P* value^●^
	mean (CI of 95%)	mean (CI of 95%)		mean (CI of 95%)	mean (CI of 95%)	
Thyroid parenchyma vascularization	2.00 (1.56–2.44)	1.87 (1.49–2.25)	0.466	1.75 (1.26–2.34)	2.30 (2.01–2.59)	0.015*

LLLT: low-level laser therapy; CI: confidence interval; ^●^: paired Student's *t*-tests were used for compare the results pre- and after intervention for each group; *: *P* < 0.05.
